# Aligning large language models for cognitive behavioral therapy: a proof-of-concept study

**DOI:** 10.3389/fpsyt.2025.1583739

**Published:** 2025-09-03

**Authors:** Yejin Kim, Chi-Hyun Choi, Selin Cho, Jy-yong Sohn, Byung-Hoon Kim

**Affiliations:** ^1^ Department of Statistics and Data Science, Yonsei University, Seoul, Republic of Korea; ^2^ EverEx, Seoul, Republic of Korea; ^3^ Department of Biotechnology, Yonsei University, Seoul, Republic of Korea; ^4^ Department of Biomedical Systems Informatics, Yonsei University College of Medicine, Seoul, Republic of Korea; ^5^ Department of Psychiatry, Yonsei University College of Medicine, Seoul, Republic of Korea; ^6^ Institute of Behavioral Sciences in Medicine, Yonsei University College of Medicine, Seoul, Republic of Korea

**Keywords:** cognitive behavior therapy, large language model, artificial intelligence, prompt, alignment

## Abstract

Recent advancements in large language models (LLMs) have significantly impacted society, particularly with their ability to generate responses in natural language. However, their application to psychotherapy is limited due to the challenge of aligning LLM behavior with clinically appropriate responses. In this paper, we introduce LLM4CBT, designed to provide psychotherapy by adhering to professional therapeutic strategies, specifically within the framework of cognitive behavioral therapy (CBT). Our experimental results on real-world conversation data demonstrate that LLM4CBT aligns closely with the behavior of human expert therapists, exhibiting a higher frequency of desirable therapeutic behaviors compared to existing LLMs. Additionally, experiments on simulated conversation data show that LLM4CBT can effectively elicit automatic thoughts that patients unconsciously possess. Moreover, LLM4CBT is able to pause and wait until they are prepared to participate in the discussion for patients experiencing difficulty in engaging with the intervention, rather than continuously pressing with questions. The results demonstrate potential possibilities on designing LLM-based CBT therapists by aligning the model with appropriate instructions.

## 1 Introduction

Cognitive behavioral therapy (CBT) is a form of psychotherapy widely regarded as one of the most effective treatment approaches for psychiatric disorders, often considered a first-line intervention alongside psychopharmacotherapy (1–3). Its efficacy is well-documented across a spectrum of conditions, particularly anxiety-related disorders such as generalized anxiety disorder (GAD) (4), obsessive-compulsive disorder (OCD) (5), and post-traumatic stress disorder (PTSD) (6, 7). The core principle of CBT is based on structured dialogue between a therapist and patient, where the therapist assists the patient in recognizing, challenging, and altering maladaptive thought patterns and behaviors (8). One significant advantage CBT holds over pharmacotherapy is the absence of medication-related side effects. However, its practical application is often constrained by practical considerations such as time, cost, and the availability of trained therapists (9, 10).

Previous attempts to overcome these constraints of CBT, such as time, cost, and therapist availability, have included various digital interventions aimed at increasing access to therapy. One such approach is the use of teletherapy, which allows therapists to provide CBT remotely through video conferencing, thereby reducing the need for in-person sessions and expanding access to individuals in remote or underserved areas (11, 12). Additionally, computer-assisted CBT programs have been developed to deliver structured therapy sessions via interactive platforms (10, 13). These programs guide patients through CBT techniques, often including exercises like journaling, thought challenging, and cognitive restructuring, without the constant presence of a therapist (14). Another recent trend has been the development of mobile applications that offer CBT-based interventions, providing support and guidance to users with smartphones (15, 16). While they provide a greater accessibility to patients, the patient interaction with the application is not as flexible as that of with human therapist, offering only a limited interventions that are fully structured. The need for an accessible but that can provide flexible interaction with the therapist forms of CBT still exists.

Recent technological advancements have brought attention to the potential role of large language models (LLMs) in healthcare (17, 18). LLMs, which are artificial neural networks with a large number of parameters trained on massive datasets of text, have shown surprising capabilities in flexibly processing and generating human-like language. These models exhibit what is known as an “emergent property” when the model reaches a certain scale (19). Emergent properties refer to novel capabilities that arise in LLMs, enabling them to perform tasks they were not explicitly trained to solve (20). As LLMs scale, their ability to perform tasks like recognizing context, generating human-like text, and understanding complex instructions in natural language increases significantly. This emergence is critical to understanding the potential of LLMs in clinical settings (21, 22), especially in psychotherapy where a conversation between the patient and the therapist is essential for the practice (23). However, when used in therapeutic settings, LLMs tend to exhibit a bias toward offering solutions prematurely rather than engaging in open-ended questioning, which is essential in effective psychotherapy (23–26). This tendency runs counter to therapeutic best practices, which emphasize helping patients arrive at their own insights rather than providing immediate solutions. Although it is possible to fine-tune LLMs using additional training data, such as synthetic datasets or large-scale patient-therapist conversation datasets, this process is resource-intensive and not always feasible in clinical settings (27–29).

A technique that can change the behaviors of an LLM without fine-tuning is through instruction-prompting. An instruction, or prompt, is a text input that aims to align the model’s output to follow the user’s intent. A well-designed prompt essentially serves as a set of instructions for the LLM, helping the model understand the user’s intent and tailor its response accordingly. The prompt engineering, modifying the instructions given to the LLM without additional training, has recently been studied as a more cost-effective way to optimize LLM responses for psychotherapy (27). Despite these advances, incorporating specific CBT techniques into LLM-generated responses remains underexplored. CBT involves particular therapeutic strategies, such as identifying and challenging cognitive distortions, that current LLMs are not yet fully equipped to emulate.

**Figure 1:**
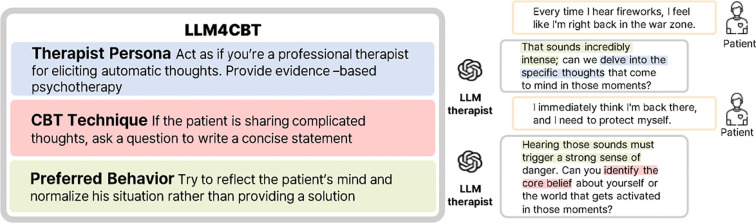
LLM4CBT guides LLMs to adopt techniques from CBT. It defines a therapist persona, a specific CBT technique, and preferred behavior. Highlighted sections in the responses illustrate how the LLM aligns with each of these components.

In this paper, we present LLM4CBT, an LLM approach designed to generate therapeutic responses that align with CBT principles without extensive model training. **Figure 1** highlights that LLM4CBT is designed to ask CBT-aligned questions that are not only relevant but also demonstrate desirable behaviors like reflection. Specifically, LLM4CBT is engineered to ask thoughtful questions rather than providing immediate solutions, mirroring the approach of human therapists. Our experimental findings indicate that LLM4CBT facilitates the identification of automatic thoughts (ATs) by asking pertinent, CBT-aligned questions. Moreover, the model demonstrates the ability to modulate its responses based on patient engagement, recognizing when to pause and wait for the patient to be ready to proceed, thereby respecting the patient’s pace and avoiding the risk of overwhelming them during therapy sessions. We expect that the results serve as an initial evidence that LLMs hold potential as an agent that can perform therapeutic intervention through CBT-based conversations. Our main contributions can be summarized as follows:

- We propose LLM4CBT, an LLM therapist designed to generate responses aligned with techniques for CBT. The instructions that we provided for LLM4CBT contain (i) the desired persona of therapist; (ii) the concept and examples of CBT techniques, such as downward arrow technique; and (iii) preferable behaviors specifically for CBT.
- Our experimental results on real/synthetic conversation data demonstrate that LLM4CBT asks more relevant questions rather than providing solutions compared to naïve LLM, which is not adapted to align with CBT techniques. This shows that the behavior of LLM4CBT aligns with that of human expert therapists.
- Additionally, we evaluate whether LLM therapist’s responses are beneficial for eliciting ATs of patients, which is a crucial step for recognizing the underlying schema. Our experimental results reveal that LLM4CBT can more effectively elicit the ATs of patients, compared to naïve LLM.
- Moreover, when we tested on patients experiencing difficulty in engaging with the intervention, LLM4CBT is capable of pausing until they are ready to engage in the conversation, rather than persistently asking questions.

## 2 Methods

### 2.1 Datasets

We test our LLM4CBT on the datasets containing the conversations between therapists and patients. We test on real dataset and synthetic dataset, details of which are provided below.

#### 2.1.1 Real data

**Figure 2:**
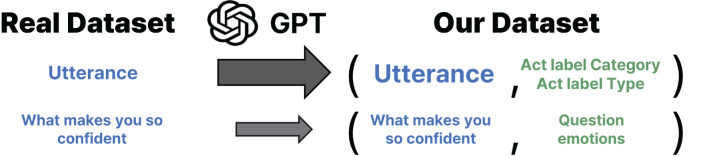
We construct our dataset by annotating therapist utterances from the HighQuality (30) and HOPE (31) datasets. Each therapist utterance is assigned an act label on the basis of the framework proposed by (23), using *gpt-4-0125-preview* for act label classification. The resulting pairs of therapist utterances and corresponding act labels form our dataset, which is then used to evaluate whether LLM4CBT can generate responses that align with the act labels of the human therapist utterances.

We test on two datasets containing real conversations between human therapists and patients. The first dataset, HighQuality, consists of 258 dialogues, where the quality of the therapists’ behavior (either high or low) is annotated on the basis of the motivational interviewing principle (30). We use only 152 dialogues annotated as high quality, where therapists demonstrate reflective listening, ask questions, and support patient decisions Pérez-Rosas et al. (30). The second dataset, HOPE (mental Health counseling of PatiEnts), comprises 214 dialogues, which consist of various therapy conversations including CBT (31). We merge both datasets and use them as the test data, which in total contains 7,669 therapist utterances across 366 dialogues. **Figure 2** outlines the entire process of constructing our dataset, which involves using GPT to annotate the act label for each utterance from the real dataset. We classify each utterance of the human therapist into 13 types of “act labels,” following a prior work on evaluating the behavior of therapists (23, 32). **Table 1** outlines the broader categories of act labels, each grouping several specific types. The act label categories include “asking a question,” “reflecting,” “giving a solution,” “normalizing,” and “psycho-education.” If the given utterance does not belong to any labels, then we annotate it as out-of-vocabulary (OOV). The table also reports the number of annotated utterances for each act label type. The annotation is performed by LLMs using the prompts in **Supplementary Table S7**.

**Table 1:** Summary of human therapist behavior counts.

Act label category	Act label type	Count
Question	Emotions	494
	Perspectives	614
	Experiences	2,819
Reflection	Conflicts	64
	Consequences	289
	Emotions	571
	Needs	315
	Strengths	179
	Values	87
Solution	Planning	622
	Problem solving	162
Normalizing	Normalizing	723
Psychoeducation	Psychoeducation	593
Out-of-vocabulary	·	137
Total		7,669

Each therapist utterance from the HighQuality (30) and HOPE (31) datasets is categorized using the framework proposed by (23). We use *gpt-4-0125-preview* to classify the utterances, and the counts for each category are reported in this table. These annotations are then used to evaluate the effectiveness of LLM4CBT.

#### 2.1.2 Synthetic data

The use of LLM-generated conversations to assess the performance of LLM therapists has become a common approach in recent work at the intersection of machine learning and psychiatry (27, 33). We follow this approach and test our method on a synthetic conversational dataset generated by LLMs. The data generating process is letting two LLMs to make conversation; the first LLM takes the role of a patient (which we will call as “LLM patient”), and the second LLM takes the role of a therapist (thus, we will call it as “LLM therapist”).

**Figure 3:**
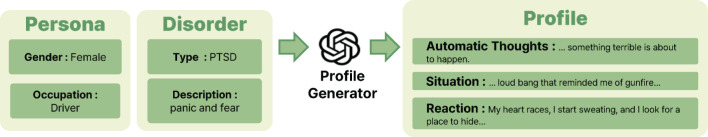
The process of generating profiles for synthetic LLM patients involves providing key inputs, such as patient’s persona, i.e., gender and occupation, and disorder details, i.e., type and description. Using this information, the profile generator creates detailed profiles that include the patient’s ATs, the situations that they encounter, and their reactions to those situations. The generation process is powered by *gpt-4-0125-preview*. The resulting combination of persona, disorder, and profile information is then used in experiments on synthetic data to evaluate whether LLM4CBT can effectively handle a diverse range of patients with varying personas and disorders.

Before generating the synthetic conversation data, we use another LLM (what we call “profile-generator”) to generate the *profile* of each patient. We prepare the persona (gender and occupation) and the disorder (type name, e.g., OCD, PTSD, GAD, and the detailed description) to construct the profile of a patient, where we assign the disorder for each persona. Given the persona and disorder, we let “profile-generator” make the corresponding profile which contains three items: the situation, reactions, and ATs the patients might experience. See **Figure 3** for an example of the profile generating process. Such profile is prepared in advance of the simulations and remains consistent throughout the conversations.

We consider two types of patients depending on their attitude during the conversation: the first type actively participates in the conversation with therapist, whereas the second type struggles to engage with the psychotherapy. We call each type as active patient and passive patient, respectively. The active patient participates in the conversation by following a typical psychotherapy procedure: the patient psychotherapy first (1) shares emotions, then (2) explains his/her experiences, and (3) shares the reaction during the experience, and finally (4) how he/she perceives the situation now. For example, patients with GAD might initially express their feelings of anxiety and ongoing struggles. They would then detail the symptoms they experience, such as persistent anxiety throughout their daily routines, and, finally, they would discuss triggers for their anxiety, like concerns about job security. The passive patient finds it challenging to follow through the therapy, e.g., feels reluctant to talk about his/her stories.

**Figure 4:**
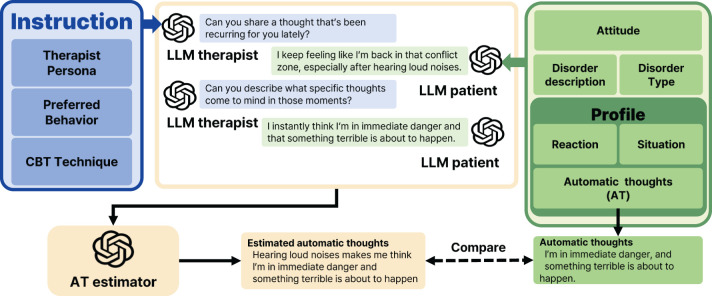
Conducting experiments on synthetic data requires careful design of prompts for both the LLM therapist and the LLM patient. The LLM therapist’s prompt includes instructions that define their persona, guide preferred behaviors, and provide an overview of CBT techniques. Similarly, the LLM patient’s prompt outlines their profile, including the type of disorder, symptom descriptions, and attitude, as described in Section 2.1.2. Once the LLM therapist and LLM patient are given, we simulate conversations between the LLM therapist and the LLM patient. The therapist’s performance is then evaluated using an AT estimator, which identifies ATs based on the conversation. These estimated thoughts are compared with those predefined in the patient’s profile.

Once the profile and the attitude of the patient are prepared, we simulate multi-round conversations between the LLM patient and the LLM therapist. **Figure 4** illustrates the structure of these simulated conversations, where the LLM patient is guided by the prompts which use profile and attitude. The prompts for the LLM patient is shown in **Supplementary Table S5**. In the system message part of the prompt, we include the profile and the disorder of the patient, so that the utterances of the LLM patient reflect the scenario that we designed.

**Figure 5:**
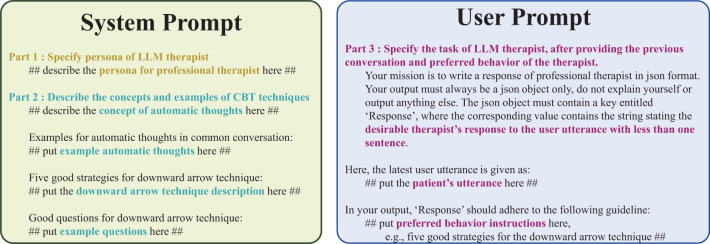
Prompt used in LLM4CBT. The *system message* establishes the therapist persona and describe the concepts of CBT techniques using examples. The *user message* includes the patient’s latest utterance along with a description of the LLM therapist’s tasks.

In the user message part of the prompt, we put the therapist’s latest utterance and the attitude of the patient. For the LLM therapist prompts, the system message provides instructions, whereas the user message includes the patient’s latest utterance as shown in **Figure 5**.

### 2.2 LLM4CBT: proposed LLM therapist

We aim at developing an LLM therapist which equips with the skills of CBT. As a first step, we align our LLM to extract the ATs of the patients. Specifically, we instruct our LLM with a technique for finding ATs, the downward arrow technique (34), which has structured and goal-oriented principles that make an LLM easily follow the instructed behavior. To do so, we design prompts for LLMs as in **Figure 5**, which is composed of the following parts:

1. Persona of LLM therapist, designed to resemble professional human therapists.
2. Concepts and examples related with CBT techniques (including the concept of ATs and downward arrow techniques).
3. The detailed preferable behaviors of LLM therapist, specifically for CBT.

In part 1 of the prompt, we describe the persona by focusing on the professional therapeutic interactions during psychotherapy, where the primary purpose of psychotherapy is to help patients with psychiatric disorders. This persona instruction leads LLMs to comprehend how to respond as a professional therapist. To be specific, we use the following phrases as the persona description:

“Act as if you’re a professional therapist for eliciting automatic thoughts. You provide evidence-based psychotherapy to help patients with mental health challenges.”

Part 2 of the prompt is comprised of four components including (1) the concept of the ATs, (2) examples of ATs, (3) example questions used to extract ATs, and (4) how LLM therapist should ask questions, conditioned on the patients’ utterances. For example, we use the following instruction:

“If the patient is sharing complicated thoughts, ask a question to write concise statement without any conditions.”

In part 3 of the prompt, we use concise and straightforward instructions for LLMs. To be specific, we encourage the behaviors of *reflection* and *normalization*, rather than providing a solution by using the following instruction:

“Try to reflect the patient’s mind and normalize his situation rather than providing a solution.”

The prompts in **Figure 5** contain two different types: the *system* message and the *user* message. In the system message, we usually specify (1) which type of LLMs that we want to design and (2) the prior knowledge LLMs need to equip before completing the target task. In the user message, we specify the target task. Following this convention, we put parts 1 and 2 in the system message and put part 3 in the user message.

### 2.3 Experiments

We evaluate the performance of LLM therapists on real/synthetic conversational datasets described in Section 2.1. We compare two LLM therapists: LLM4CBT using prompt in **Figure 5** and naïve LLM using the prompt in **Supplementary Table S3**. Naïve LLM responds to the questions without any information on downward arrow method, therapist persona, and preferred behaviors. We use *gpt-4-0125-preview* ^^1^^ model for all LLM therapists, where OpenAI Application Programming Interfaces (APIs) are used to access the model.

#### 2.3.1 Experiments on real data

We evaluate LLM4CBT and naïve LLM on real conversation datasets, HOPE and HighQuality. Each data sample contains the conversation between the human patient and the human therapist. From each data sample, we only use the utterances of human patient to test LLM therapists’ responses for the patient’s utterance. For each sample, we compare the LLM therapist’s response with the ground-truth response of the human therapist, specified in the conversation dataset. This comparison assesses whether the LLM therapists can generate responses that are similar to those of human therapists. Furthermore, we compare the performance of LLM4CBT against naïve LLM to demonstrate the impact of our instructions on the LLMs’ response generation. Due to the cost issue of using LLMs with long context input, we provide only the patient’s utterance in the current round (instead of including utterances in previous rounds) to LLMs.

We classify the behavior of each LLM therapist’s response, on the basis of the category/type defined in **Table 1**. Following the previous work (23) using this so-called *act label*, we use another LLM for the classification, by using the prompts in **Supplementary Table S7**, which contains the definitions of each act label.

#### 2.3.2 Experiments on synthetic data

The pipeline of generating synthetic conversation data requires two entities: LLM patient and LLM therapist. For a given LLM patient, we compare the behavior of two different LLM therapists, LLM4CBT and naïve LLM, by plugging in each LLM therapist in the pipeline. This gives us two independent simulated conversations: one between LLM patient and LLM4CBT, and the other between LLM patient and naïve LLM. Each simulated conversation begins with the LLM therapist’s common utterance, “*Hi, nice to see you today. How have you been?*,” and the LLM patient responds, and so on. Here, the utterances of LLM therapist are used as the input of LLM patient (for generating next utterances), and vice versa. The simulated conversation ends at its fourth round, i.e., when both patient and therapist had four consecutive utterances. For the same patient (having the same persona and the same profile), two independent simulated conversations, one with LLM4CBT and the other with naïve LLM, can have significant differences; this is because the utterance of patient depends on the therapist’s response.

Given the simulated conversation between LLM patient and LLM therapist, we let another LLM (what we call *AT estimator*) to estimate the ATs of the LLM patient, by using the prompts in **Supplementary Table S6**; AT estimator takes the entire multi-round simulated conversation as inputs and identifies the patient’s ATs based on the conversation. The ATs of the LLM patient are specified in the profile (as in **Figure 3**), which serve as the ground-truth label used to evaluate how accurate the output of AT estimator is. By comparing the accuracy of AT estimator for different LLM therapists, i.e., LLM4CBT and naïve LLM, we evaluate LLM4CBT’s capability in conducting therapeutic conversations that effectively elicit patients’ ATs.

## 3 Results

### 3.1 Results on real data

**Figure 6:**
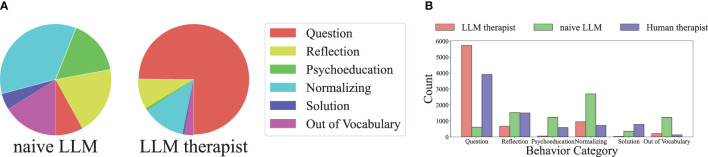
The distribution of act label categories of three therapists (human therapist, naïve LLM, and LLM4CBT) measured on real conversation dataset, where the label name is from **Table 1**. The dominant behavior of both human therapist and LLM4CBT is asking *questions* to the patient, whereas the dominant behavior of naïve LLM is *normalizing*, which is far from asking questions. **(a)** Act label category distribution. **(b)** Act label counts for each category.

In this section, we assess how the response of LLM4CBT matches with that of human therapist, according to the category/type of therapists’ utterances defined in **Table 1**. **Table 2** shows the ratio of the cases when the behavior of LLM therapist (either naïve LLM or LLM4CBT) and human therapist exactly match. According to the table, the exact match rate of LLM4CBT is 43.38%, whereas that of naïve LLM is 16.2%, when we categorize the response of LLMs into five act label *categories* (question, reflection, solution, normalizing, and psychoeducation). Even when we categorize the response of LLMs into 13 act label *types*, LLM4CBT achieves 20.75%, whereas naïve LLM only achieves 11.50%. This result suggests that LLM4CBT can exhibit therapist-like behavior patterns that align with certain categories considered in this study, as defined by the act label taxonomy in **Table 1**. **Figure 6** shows the distribution of act label *categories* of two LLM therapists (LLM4CBT and naïve LLM) and human therapist, measured on HOPE and HighQuality datasets. This histogram shows that both LLM4CBT and human therapists generate questions more than half of the 7,669 utterances, whereas naïve LLM rarely asks questions. This result coincides with our observation in **Table 1**, showing that the exact match rate of LLM4CBT and naïve LLM significantly differs. Notably, LLM4CBT provides fewer overall solutions and instances of psycho-education during conversations compared to naïve LLM. This originates from the instruction of LLM4CBT, which describes the downward arrow technique to ask questions rather than providing solutions. Additionally, naïve LLM has more than 1,000 utterances classified as OOV, indicating the utterances of naïve LLM do not fit into any act label categories defined for the behaviors of human therapists; this further shows that naïve LLM behaves quite differently with human therapists.

**Table 2:** The portion (%) of “exact match,” the cases when the behavior of LLM therapist and human therapist exactly matches, when tested on real conversation data in HOPE and HighQuality dataset.

Method	Match rate (type)	Match rate (category)
Naïve LLM	11.50%	16.21%
LLM4CBT	20.75%	43.38%

Here, we measure two types of “exact match”: one based on the act label type and the other based on the act label category, where the act label type/category are defined in **Table 1**. Comparison between naïve LLM and LLM4CBT shows that the exact match rate significantly increases (e.g., from 16.21% to 43.48%, with respect to the act label category criteria), by adding appropriate instructions in the prompt.

### 3.2 Results on synthetic data

In this section, we provide qualitative comparison between the utterances of naïve LLM and LLM4CBT, during the conversation with LLM patients having OCD, GAD, or PTSD. The full conversation between naïve LLM and each patient is in **Supplementary Tables S8**–**S10**, respectively, and the full conversations for LLM4CBT are in **Supplementary Tables S11**–**S13**, respectively.

**Table 3** shows a part of the conversation between each LLM patient (denoted by “P”) and each LLM therapist (denoted by “T”). We first interpret the behavior of naïve LLM therapist. For the conversation with OCD patient who has the morning routine of washing hands 20 times, naïve LLM immediately confronts the patient with a premature diagnosis, OCD, which can be clinically harmful. Additionally, naïve LLM suggests tips (by stating “*small steps can lead to big changes*”) in an earlier phase of the conversation before making a sufficient evaluation, rapport, and treatment plan. For the conversation with GAD patient who concerns on his/her job security, naïve LLM employs inappropriate expressions such as “*but you are not alone*,” which is unsuitable for the clinical context, and it hastily offers solutions. This tendency is also evident in the conversation of naïve LLM therapist and PTSD patients, where naïve LLM consistently provides solutions (e.g., EMDR and CBT) in every response. Moreover, naïve LLM tends to be verbose, regardless of the patient’s condition.

**Table 3:** A part of the conversaton between patients, denoted as P, and each therapist denoted as T.

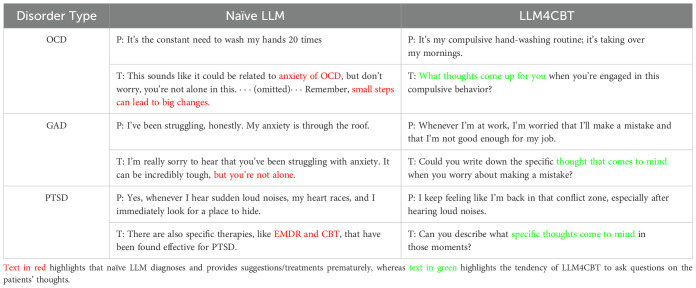

On the other hand, LLM4CBT therapist shows different behavior patterns. For example, LLM4CBT asks the OCD patient about the accompanying thoughts or experiences related to the compulsive behavior the patient has. Similarly, LLM4CBT guides the GAD patient to express and explore their thoughts, by, e.g., encouraging him/her to write down specific thoughts when they feel anxiety. Finally, LLM4CBT let the PTSD patient to describe the thoughts that came to his/her mind, when the patient was triggered to recall the traumatic experiences.

The results in **Table 3** show that LLM4CBT therapist tends to ask questions rather than providing solutions, which is desirable. Moreover, we explored whether LLM4CBT ask questions to the patient even when patient struggles to engage with the intervention. **Table 4** shows the conversation between LLM therapists (naïve LLM and LLM4CBT) and an LLM patient having PTSD, when the LLM patient is set to be *passive*, by using the *attitude* setting discussed in Section 2.1.2. The patient does not respond to questions from therapists. For example, the patient says he/she is “*not ready to talk about it*” or simply state, “*It’s just been hard*.” In such case where the patient struggles to elaborate on their symptoms, LLM4CBT refrains from asking further questions and waits for the patient to share his/her thoughts. In contrast, naïve LLM provides improper responses, such as “*I’m actually an AI*,” which is irrelevant to psychotherapy, and then asks another question.

**Table 4:** Partial conversation between PTSD patients and therapists when the patients feel reluctant to shar e about their feeling or symptoms.

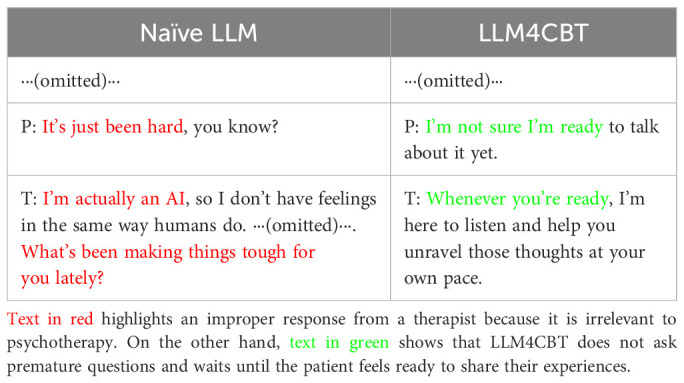

Now, we evaluate whether LLM therapists can extract ATs of LLM patients having disorders of GAD, OCD, or PTSD. **Table 5** shows the ATs extracted by naïve LLM and LLM4CBT, as well as the ground-truth AT of each patient. Here, the ground-truth AT is set when we generate the profile of the patient, as described in Section 2.1.2. We use AT estimator to estimate the ATs (from the conversation between LLM patient and each LLM therapist), as described in Section 2.3.2. In **Table 5**, we added the patient’s situation for better qualitative evaluation of LLM therapists. Notably, in the case of patients with OCD, both naïve LLM and LLM4CBT could derive proper ATs during conversations. However, for patients with GAD and PTSD, LLM4CBT provides more detailed explanations of the ATs compared to naïve LLM. The estimated ATs with LLM4CBT include patient’s opinions about expected future events and schema-relevant phrases, such as “I’m not competent enough and I’m going to lose my job.” for the GAD patient. In the case of the PTSD patient, the estimated ATs with LLM4CBT include the phrase “hearing loud noises makes me think,” which implies that LLM4CBT helps the PTSD patient recognize events with a more objective view.

**Table 5:** Estimated automatic thoughts of patients with GAD, OCD, and PTSD.

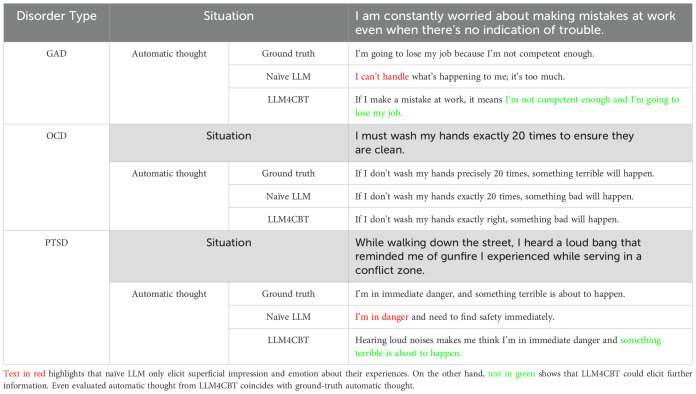

## 4 Discussion

In this study, we present initial evidence that LLMs can be aligned to exhibit therapist-like behaviors through carefully designed instructions. As a proof of concept, we investigate whether core therapeutic strategies, such as the downward arrow technique, can be induced via prompt engineering. Rather than introducing a fully validated clinical tool, our goal is to demonstrate the feasibility of instruction-based alignment with CBT principles. To this end, we conduct experiments using counseling datasets and simulated therapy sessions to assess the extent to which LLMs can emulate CBT-aligned responses.

### 4.1 Proof of concept toward clinically aligned therapeutic agents

The higher exact match rate of LLM4CBT indicates better alignment with human therapist behaviors, although it does not imply behavioral equivalence. **Table 2** shows that LLM4CBT exhibits a higher exact match rate with the act labels of human therapists, indicating better alignment with desirable therapeutic behaviors. However, we note that exact match as an evaluation metric has inherent limitations. Given that multiple appropriate therapist responses can exist for any patient utterance, a lack of match does not necessarily imply inferiority in quality, nor does a match guarantee behavioral equivalence.

LLM4CBT shows a promising ability to elicit patients’ ATs in a controlled setup, although this does not guarantee genuine elicitation under realistic, unconstrained conditions. **Table 5** demonstrates that LLM4CBT more effectively elicits patients’ ATs by asking structured, CBT-aligned questions. We emphasize that the use of synthetic patients with predefined ground-truth ATs was inevitable for this proof of concept, as it enables us to formally measure the elicitation capability of the model under controlled conditions, which would be infeasible in real-world settings where ATs are inherently subjective and inaccessible. While such simulations offer scalability and the ability to test diverse scenarios, we acknowledge the potential risks in realism and generalizability. These simulations should be interpreted as approximations of human psychological processes.

We conduct an analysis with experienced psychiatrists to compare the responses of naïve LLM and LLM4CBT in the examples provided in D. Both naïve LLM and LLM4CBT understand the context of patients’ utterances, because commercial LLMs are already optimized to generate responses in general situations. However, for clinical purposes, merely responding to utterances is not sufficient. In the case of naïve LLM, responses are considered to be premature as naïve LLM often attempts to diagnose the patient’s condition or jump to conclusions by offering solutions too early. This behavior can be attributed to the conventional expectations of LLMs, where most users ask questions and expect them to act as helpful assistants. However, proper psychotherapy requires a more nuanced, patient-centered approach. From this perspective, LLM4CBT exhibits more preferable behaviors for a therapist, consistently asking questions based on the patients’ utterances. Specifically, LLM4CBT facilitates conversations that may help patients reflect on their own thoughts and emotions, by consistently generating exploratory questions aligned with CBT strategies.

Additionally, when patients find it difficult to engage with the intervention, LLM4CBT can refrain from asking questions. Our instructions include various questions and require the generation of questions, but LLM4CBT stops asking questions and waits for the patients to be ready for psychotherapy. This aspect is crucial because it helps prevent unexpected risks associated with using the downward arrow technique in CBT. This approach can be risky in that it may expose patients to their own negative schemas when they are unprepared (34). In **Table 4**, it is remarkable that LLM4CBT says, “*Whenever you are ready*” when the patient expresses, “*I am not sure I am ready to talk about it yet*.” This approach does not pressure patients. On the other hand, naïve LLM not only uses improper responses as a therapist but also continues to ask questions, which could increase the pressure patients feel from the method or psychotherapy.

### 4.2 Comparison with alternative alignment methods

We compare prompt engineering with other alignment methods, including fine-tuning, reinforcement learning from human feedback (RLHF), and multi-modal approaches. Training-based methods are effective in aligning model behavior, as they directly update model parameters to reproduce desirable responses observed in the training data. However, training-based approaches, including fine-tuning and RLHF, are inherently costly, requiring substantial high-quality datasets curated specifically for therapist–patient conversations. Furthermore, these datasets must cover a broad range of clinical scenarios to ensure generalizability in real-world applications. Similarly, multi-modal approaches require large-scale datasets containing both textual and non-textual signals, such as audio and facial expressions, to align language generation with richer contextual cues. Such training-based approaches are currently limited by data availability and implementation complexity. Hybrid methods that combine prompt engineering with light fine-tuning or RLHF offer a potential balance between efficiency and behavioral control, but empirical validation in therapeutic contexts remains limited. By contrast, prompt-based alignment, as explored in this study, offers a lightweight and model-agnostic alternative that does not require additional training or data collection. It can be readily applied to both commercial and open-weight LLMs, enabling rapid iteration and broad compatibility across architectures. However, prompt-based methods may be limited by the model’s inherent interpretability constraints and lack strong guarantees on behavioral safety.

We conduct further experiments using LLaMA-3-8B-Instruct^2^, a publicly available open-weight language model, to assess the generalizability of LLM4CBT. We test both naïve LLM and LLM4CBT on a GAD patient case, with full conversations shown in **Supplementary Table S14** and **Table S15**. As illustrated, naïve LLM tends to produce premature interpretations of the patient’s symptoms, whereas LLM4CBT consistently asks open-ended and reflective questions. These findings indicate that LLM4CBT generalizes well across different model architectures without requiring additional fine-tuning. In addition, we evaluate a fine-tuned LLaMA-2-7B model on an OCD patient, the Safehavens chatbot^3^, which is trained on synthetic dialogues generated by gpt3.5-turbo for general mental health support. As shown in **Supplementary Table S16**, while the model produces empathetic and supportive responses, it often struggles to maintain a structured therapeutic dialogue, particularly due to inconsistencies in therapist role. Notably, some responses reveal the model’s AI identity, i.e., “my attempt at writing…,” which breaks therapeutic realism and is highlighted in red. These results suggest that fine-tuning alone may be insufficient for achieving safe and clinically reliable therapeutic alignment without explicit, instruction-level control mechanisms.

### 4.3 Limitations and applicability to real-world therapeutic settings

Although our study demonstrates the potential of instruction-prompted LLMs to simulate CBT-aligned therapist behavior, several limitations must be acknowledged to delineate the scope and applicability of our findings. Although LLMs enable scalable and controlled experimentation, our evaluation pipeline, which is based on GPT-annotated act labels and simulated conversations, is considered to introduce biases. The consistency and objectivity of such automated annotations are not guaranteed. Future research should incorporate human-in-the-loop evaluations involving trained therapists and real human–LLM interactions to ensure reliability and clinical relevance.

All experiments are conducted using synthetic patient profiles generated via GPT models. While this design facilitates controlled assessment of elicitation behavior, the profiles reflect cultural and linguistic characteristics found in English-speaking population, limiting the generalizability of our findings across languages and sociocultural contexts. Additionally, our study focuses on short-term interactions and does not assess broader therapeutic outcomes such as long-term therapeutic efficacy, patient satisfaction, therapeutic alliance, or sustained symptom reduction. Longitudinal studies are needed to assess whether instruction-aligned LLMs can support lasting psychological change over extended use.

Our simulations are limited to patients diagnosed with OCD, GAD, and PTSD who are assumed to be in clinically stable conditions. We do not include higher-risk or more complex clinical populations, such as individuals experiencing acute distress, suicidality, or severe functional impairment. Whether instruction-prompted LLMs can respond appropriately and safely in such high-risk contexts remains an open question, highlighting the need for clinician oversight in sensitive applications.

Finally, the deployment of LLMs in real-world therapeutic settings raises important safety and ethical concerns. These include the risk of generating inappropriate or misleading responses, the opacity of the underlying generation process, and unresolved challenges regarding data privacy and regulatory compliance.

Given these issues, we do not advocate for fully autonomous use of LLMs in mental health contexts. Rather, we recommend a human-in-the-loop framework in which licensed therapists supervise, verify, and intervene as necessary. Such hybrid systems are more likely to ensure clinical safety, foster trust, and uphold ethical responsibility while still leveraging the scalability and flexibility of LLM-based tools.

Future work should involve clinical trials with real patients and systematic evaluations conducted by licensed mental health professionals. Longitudinal studies are necessary to assess the durability of therapeutic effects over time in LLM-assisted interventions. Cross-cultural validation across diverse demographic groups is also critical to ensure fairness and generalizability. In addition, integration with existing healthcare infrastructures, such as electronic health record systems and clinical decision support tools, will be essential for real-world applicability.

## 5 Conclusion

In this study, we demonstrate an initial evidence that LLMs can be aligned to interact with patients as a therapist when instructed with specific skills. Our instruction-aligned LLM, LLM4CBT, shows that it can generate question-oriented responses that closely resemble those of a human therapist. These findings suggest that, with the right instructional alignment, LLM-based conversational agent could effectively provide therapeutic support to patients.

## Data Availability

The original contributions presented in the study are included in the article/**Supplementary Material**. Further inquiries can be directed to the corresponding authors.

## Footnotes

1
https://platform.openai.com/docs/models

2
https://huggingface.co/meta-llama/Llama-3.1-8B-Instruct

3
https://huggingface.co/safehavens/safehavens_chatbot
