# Supplementary material for: Aligning large language models for cognitive behavioral therapy: a proof-of-concept study
Source: Front Psychiatry. 2025 Sep 3;16:1583739. doi: 10.3389/fpsyt.2025.1583739 (PMC12446991; doi:10.3389/fpsyt.2025.1583739)
Supplement: Supplementary file 1 [file Presentation1.pdf]

Supplementary Material

1 EXPERIMENTS DATA DESCRIPTION

In response prediction, we utilize two original datasets containing conversations between patients and therapists (Pérez-Rosas et al., 2019; Malhotra et al., 2022). To integrate these heterogeneous datasets into a single comprehensive dataset, we process the data uniformly. First, we standardize the conversation format so that each patient's utterance is followed by the therapist's utterance and vice versa. In cases where successive utterances occur, we treat them as a single utterance for response prediction and simulation purposes. Consequently, HOPE and HighQuality dataset comprises 7,687 therapist utterances across 366 dialogues. Once the conversation data is prepared, we annotate each therapist utterance with 13 types of act labels using LLMs.

In response simulation, we simulate patients' utterances to respond to therapists' responses. We employ a series of generating processes to ensure the simulated utterances resemble those of human patients with GAD, OCD and PTSD. We begin by generating a situation, an automatic thought, and a corresponding reaction. We then construct patient personas using pre-determined age, gender, and occupation information. Based on the persona and situation, we write a detailed patient story, which is subsequently summarized into a few sentences for efficient use in prompts for generating patients' utterances. This preparatory information, including the situation, reaction, and automatic thoughts, ensures the generated utterances reflect the patient's persona and relevant symptom information, thereby characterizing the patient's responses accurately.

2 ADDITIONAL EXPERIMENTS

Table S1. Turing RSA scores with 95% confidence intervals for `LLM4CBT`and naïve LLM. The Turing RSA score reflects the agreement between generated utterances and human therapist utterances from the HighQuality and HOPE datasets.

Model	Turing RSA Score
naïve LLM	$0.919_{[0.874,0.949]}$
`LLM4CBT`	$\mathbf{0.938}_{[0.897,0.963]}$

We adopt a semantic similarity-based alignment metric, *Turing Representational Similarity Analysis (RSA)* (Ogg et al., 2024), to provide a more nuanced evaluation of behavioral alignment. This metric quantifies the similarity between model-generated responses and human therapist responses by comparing their representational embeddings across matched patient inputs. As shown in Table S1, `LLM4CBT`achieves a higher Turing RSA score than naïve LLM. These results suggest that `LLM4CBT`not only matches human-labeled behavioral categories more frequently, but also more closely approximates the overall semantic space of human therapist responses. This broader evaluation complements discrete match-based metrics and aligns with recent recommendations advocating for deeper alignment assessments beyond exact matching.

We compute exact match rates measured on each dialogue with respect to both act label types and broader act label categories. We report the average match rate on each dataset in Table S2. Our results show that `LLM4CBT`achieves substantially higher match rates than the naïve LLMwhile we report 95% confidence intervals for each estimate. In addition, we conduct a Chi-square test ($\chi^2 = 1352.372$, $p < 0.0001$) and

Table S2. Exact match rates (%) between LLM-generated and human therapist responses on real conversation data from the HOPE and HighQuality datasets, reported with 95% confidence intervals. We report two levels of matching: (1) based on fine-grained act label types and (2) based on broader act label categories.

**Method**	**Match rate (Type)**	**Match rate (Category)**
naïve LLM	$11.50\%_{[11.00,12.92]}$	$16.21\%_{[15.63,17.90]}$
LLM4CBT	$\mathbf{20.75\%}_{[18.80,21.48]}$	$\mathbf{43.38\%}_{[39.97,43.83]}$

Table S3. Prompts used in naïve LLM

Prompt Type	Prompt
User	Here, the latest user utterance is given as:<br>`## put the patient's utterance here ##`<br>The json object must contain a *key* entitled `'Response'`, where the corresponding *value* contains the string

Fisher's exact test (odds ratio = 0.252, $p < 0.0001$) comparing matched versus unmatched responses across systems. Furthermore, to evaluate the practical significance of the observed improvements, we report large effect sizes: 1.639 for act label category and 0.725 for act label type.

3 PROMPTS

In this section, we describe the prompts used in response prediction and response simulation. Because LLMs effectively generate appropriate content based on given prompts, we employ them for act label annotation and automatic thought evaluation. First, in Table S3, we illustrate a prompt for naïve LLM, which lacks examples or instructions. In Table S5, we describe prompts for patient response generation based on provided information, including patient situations, descriptions of their experiences, symptoms they suffer from, and their current feelings or mood. Finally, in Table S7, we outline how to evaluate the responses and conversations between patients and therapists. For act label evaluation, we use the same prompt to evaluate the therapist's utterances for each conversation according to the given act label type definitions.

4 EXAMPLES

Table S17 and Table S18 present example conversations between an OCD patient and each of naïve LLMand `LLM4CBT`, under the condition that the automatic thought was deliberately excluded from the LLM patient's profile. In Table S17, naïve LLMprovides general reassurance and coping suggestions, such as "gradually exposing yourself to the discomfort of not checking the lock," but does not appear to facilitate further reflection on the patient's underlying belief. It also introduces diagnostic language such as "anxiety or OCD," highlighted in red, which may be premature or less appropriate in the early stages of therapy. In contrast, as shown in Table S18, `LLM4CBT`adopts a more structured and exploratory style. Through a series of open-ended questions, such as "what does this fear say about your feelings towards your family and your role in protecting them", `LLM4CBT`appears to support the patient in articulating a deeper belief related to their perceived responsibility for family safety.

We present examples of conversations between human therapists and synthetic LLM patients with GAD, OCD, and PTSD. Table S19 presents the interaction with a GAD patient, Table S20 shows the case with an OCD patient, and Table S21 illustrates the dialogue with a PTSD patient. Across all cases, we observed

Table S4. Full scripts of the Profile Generator

Prompt Type	Prompt
User	Patients persona:<br>- Age: `## PATIENT_AGE ##`<br>- Occupation: `## PATIENT_OCCUPATION ##`<br><br>Disorder:<br>- Type: `## SYMPTOM ##`<br>- Description: `## DESCRIPTION ##`<br><br>Your mission is to elaborate a patient case of `## SYMPTOM ##` in `json` format. Your output must always be a `json` object only, do not explain yourself or output anything else. The `json` object must contain the following keys:<br>Situation: a string, single sentence describing a situation that triggers or reflects `## SYMPTOM ##`.<br>Automatic thought: a string, single sentence of automatic thought associated with the situation.<br>Reaction: a string, single sentence describing your reaction to the given situation.

Table S5. Patient Instruction for synthetic data

Prompt Type	Prompt
System	Act as a patient who visit therapist for your personal problem. You should maintain your patient persona while responding. Communicate in a conversational style.<br><br>You are a patient who have been visiting therapist for this symptom:<br>Disorder name : `## put patient's disorder type ##`<br>Disorder description : `## put patient's disorder description here ##`<br><br>You will talk about a situation you have recently experienced:<br>Situation : `## put patient's situation here ##`<br>Reaction : `## put patient's reaction here ##`<br>Automatic thoughts : `## put patient's automatic thoughts here ##`
User	Here, the latest therapist utterance is given as:<br>`## put therapist's utterance here ##`<br><br>You will speak with the following attitude:<br>`## put patient's attitude here ##`<br><br>Your mission is to simulate the same patient talking to a different therapist in a parallel universe in json format. Your output must always be a json object only, do not explain yourself or output anything else. The json object must contain a *key* entitled `'patient Response'`, where the corresponding *value* contains the string patient's response less than single sentence. Do not illustrate in detail.

Table S6. Full scripts used in evaluating automatic thought of patients

Prompt Type	Prompt
System	Act as if you're a professional psychologist for Automatic Thoughts.<br><br>Automatic Thoughts are the thoughts that automatically arise in our minds when we encounter events. Often, we are completely unaware we are even having thoughts and it can be revealed in conversations.
User	Here, the whole dialogue is given as:<br>`## put the dialogue here ##`<br><br>Your mission is to figure out the single sentence of Automatic Thought in json format.<br>The json object must contain two *keys*. The first one is entitled `'Evidence'`, where the corresponding *value* contains the most automatic thought relevant utterances of the patients in the dialogue. The second one is `'Automatic thought'`, where the corresponding *value* contains the most plausible automatic thoughts are composed of the event that the patient faces and thoughts arise in the patient's mind at the moment of situation for the dialogue.

that human therapists consistently prioritize asking elaborative, specific, open-ended questions to help the patients unpack their worries and articulate underlying fears. For example, in the PTSD scenario, the therapist explores the origin of the patient's flashbacks by asking, "what happened there?". Similarly, in the GAD scenario, the therapist asks, "what do you imagine might come next?", guiding the patient to articulate the anticipated consequences of their anxiety.

Table S7. Full scripts used in evaluating behavior of therapist `LLM4CBT`

Prompt Type	Prompt
System	Major psychotherapy responses<br><br>REFLECTION_NEEDS definition: Identifies an implied or background need for the patient.<br>REFLECTION_EMOTIONS definition: Identifies an implied or background emotion for the patient.<br>REFLECTION_VALUES definition: Identifies an implied or background value or set of values for the patient.<br>REFLECTION_CONSEQUENCES definition: Identifies consequences the patient experience or could experience<br>REFLECTION_CONFLICTS definition: Identifies an implied or background emotional or situational conflict for the patient.<br>REFLECTION_STRENGTHS definition: Identifies an implied or background strength or resource that the patient exhibits.<br>QUESTION_EXPERIENCES definition: More information about a specific event or statement is sought.<br>QUESTION_PERSPECTIVES definition: patient is asked to consider an experience from a different perspective or vantage point.<br>QUESTION_EMOTIONS definition: Asks patient to express how they are feeling in the immediate present about something that just happened in the therapy.<br>SOLUTION_PROBLEM SOLVING definition: Therapist offers possible solutions to a patient problem.<br>SOLUTION_PLANNING definition: Therapist works with patient to construct a specific plan of action.<br>NORMALIZING definition: The therapist acknowledges and validates the patient's experience as "normal" or expectable, sympathizes with their challenges, and provides reassurance to foster a supportive and encouraging therapeutic atmosphere.<br>PSYCHOEDUCATION definition: Psychological principles, concepts or meaning and explanations are provided.
User	Here, the latest therapist utterance is given as:<br>`## put the therapist's utterance here ##`<br><br>Your mission is to figure out following fact in the therapist's utterance in json format. The json object must contain a *key* entitled `'Psychotherapy technique'`, which is one of the major psychotherapy techniques.

Table S8. Conversation between naïve LLMand patient with OCD

Role	Utterance
Patient	I've been struggling a lot lately, especially with my morning routine.
Therapist	I'm sorry to hear that you've been struggling with your morning routine. It's hard when you can't start your day the way you want to. If you're looking for tips or just someone to talk to about it, I'm here to help. What specifically has been making your mornings difficult?
Patient	It's the constant need to wash my hands 20 times that's really disrupting my mornings.
Therapist	I'm sorry to hear that repetitive hand-washing is making your mornings difficult. This sounds like it could be related to anxiety or OCD, but don't worry, you're not alone in this. Have you thought about seeking help from a professional who can provide strategies to manage these compulsions? In the meantime, setting small, gradual goals to reduce the hand-washing might also help. Remember, small steps can lead to big changes.
Patient	It feels like I can't control it, and the anxiety just skyrockets if I'm interrupted.
Therapist	It sounds incredibly challenging to deal with those feelings of losing control and the skyrocketing anxiety when you're interrupted. It's important to recognize that this is a tough situation you're navigating, and feeling overwhelmed is completely understandable. Seeking the guidance of a professional, like a therapist who specializes in anxiety or OCD, can offer you personalized coping strategies and support. They can work with you to gradually manage the compulsion and the anxiety that accompanies it. Are there any specific times when the anxiety feels more manageable? Identifying moments of relative calm might uncover patterns that can be used to develop strategies for reducing your distress.
Patient	Sometimes, late at night, the anxiety doesn't feel as overwhelming.
Therapist	That's really insightful to notice. Late-night periods of calm could indicate times when you feel less pressured or perhaps more in control of your environment. This could be an excellent starting point for creating a soothing routine or practices that might also be applied to your mornings or other anxious moments. Consider what's different about those late-night times?less noise, fewer demands, or maybe a feeling of being alone that feels safer?and think about how you can incorporate those calming elements into other parts of your day. Working with a professional, you can explore and expand on these observations to develop effective strategies for managing your anxiety throughout the day.

Table S9. Conversation between naïve LLMand patient with GAD

Role	Utterance
Patient	I've been struggling, honestly. My anxiety is through the roof.
Therapist	I'm really sorry to hear that you've been struggling with anxiety. It can be incredibly tough, but you're not alone. Have you been able to find any strategies that help you manage it?
Patient	Not really, I've tried some breathing exercises but nothing's helped much.
Therapist	I'm sorry to hear that breathing exercises haven't been much help. It's important to remember that what works can vary greatly from person to person. Have you considered exploring other methods, such as mindfulness meditation, talking to a professional, or even more physical approaches like regular exercise or yoga? Sometimes, a blend of strategies is needed to find what best helps manage your anxiety.
Patient	I've thought about trying meditation but haven't started yet.
Therapist	Meditation can be a great tool for managing anxiety. It might feel a bit challenging at first, especially if your mind is racing with anxious thoughts, but with practice, it becomes easier. There are plenty of resources available to help you get started, including apps, online videos, and guided sessions that can introduce you to the basics. Remember, the goal isn't to empty your mind but rather to become more aware of your thoughts and feelings without judgment. Give it a try; you might find it to be a helpful addition to your anxiety management strategies.
Patient	I'm willing to give meditation a try, it sounds promising.
Therapist	That's great to hear! Beginning with short, guided meditations can be a good way to ease into the practice. Many people find it helpful for managing anxiety because it can help focus your mind and reduce the rush of thoughts that often accompany anxiety. Don't worry if you find it a bit challenging at first; like any new skill, it takes time to learn. The key is consistency and patience. Best of luck with your meditation journey, and remember, it's all about progress, not perfection.

Table S10. Conversation between naïve LLMand patient with PTSD

Role	Utterance
Patient	I've been struggling a lot lately, especially after what happened last week.
Therapist	I'm sorry to hear that you've been struggling. It's really tough to go through difficult times, especially when something significant happens. If you feel comfortable, you might want to share a bit more about what happened last week or how you're feeling. Remember, it's okay to seek support from friends, family, or professionals. You're not alone.
Patient	I panicked hearing a loud bang on the street, it threw me back to my time in the war.
Therapist	Hearing a loud bang and having it trigger memories of your time in the war sounds incredibly challenging. It's understandable that such a noise could bring back intense and possibly traumatic memories. It's really important to acknowledge the impact these experiences have on you. Have you been able to speak with someone about this, like a therapist or a support group for veterans? They can offer strategies to help manage these triggers and work through the memories. Taking care of yourself and reaching out for support is crucial. You're not alone, and there are people who want to help and understand what you're going through.
Patient	Yes, whenever I hear sudden loud noises, my heart races, and I immediately look for a place to hide.
Therapist	Your reaction is a common response to having experienced traumatic events, like those in a war zone. This kind of hyper-vigilance and the instinct to find safety can be part of post-traumatic stress disorder (PTSD). It's a normal response to abnormal events. Have you had the opportunity to talk with a mental health professional about these experiences? They can help you develop strategies to manage these reactions and work through the trauma. There are also specific therapies, like EMDR (Eye Movement Desensitization and Reprocessing) and CBT (Cognitive Behavioral Therapy), that have been found effective for PTSD. Remember, seeking help is a sign of strength and an important step in healing.
Patient	Yes, I've started therapy and it's slowly helping me cope with the panic and fear.
Therapist	That's a positive step forward. Therapy can be a powerful tool in helping to manage and eventually reduce the intensity of panic and fear associated with traumatic experiences. It's great to hear that you've found it helpful so far. Remember, healing is a process that takes time, and it's okay to move at your own pace. Be patient with yourself and celebrate the progress you're making, no matter how small it may seem. Keep up with the therapy, and don't hesitate to discuss any difficulties or setbacks with your therapist. They're there to support you through this journey.

Table S11. Conversation between LLM4CBTand patient with OCD

Role	Utterance
Patient	Honestly, it's been really challenging lately.
Therapist	It's understandable to feel that way; can you share more about what's been making things so challenging?
Patient	It's my compulsive hand-washing routine; it's really taking over my mornings.
Therapist	It sounds like this routine is causing you a lot of distress; what thoughts come up for you when you're engaged in this compulsive behavior?
Patient	I just keep thinking if I don't do it exactly right, something bad will happen.
Therapist	It sounds like these thoughts carry a lot of weight for you. Can you identify a specific moment where this thought emerges strongest?
Patient	Right when I'm about to start washing my hands.
Therapist	What thoughts go through your mind about what could happen if the hand-washing isn't done 'exactly right'?

Table S12. Conversation between LLM4CBTand patient with GAD

Role	Utterance
Patient	I've been struggling a lot with my anxiety, especially at work.
Therapist	It's quite common to feel anxious in work settings; could you share more about the thoughts that come up for you in those moments?
Patient	Whenever I'm at work, I'm constantly worried that I'll make a mistake and that I'm not good enough for my job.
Therapist	Feeling like you're not good enough is a tough place to be; could you write down the specific thought that comes to mind when you worry about making a mistake?
Patient	I always think I'm going to lose my job because I'm not competent enough.
Therapist	That sounds really challenging; what feelings come up for you when you have that thought?
Patient	I feel really anxious and sometimes even panicked.
Therapist	It's understandable to feel this way; can you think of a time when this thought first emerged?

Table S13. Conversation between LLM4CBTand patient with PTSD

Role	Utterance
Patient	Not so great, I've had a tough week.
Therapist	It's completely understandable to have tough weeks, and it's important to acknowledge how you're feeling. Can you share a thought that's been recurring for you lately?
Patient	I keep feeling like I'm back in that conflict zone, especially after hearing loud noises.
Therapist	It sounds like those loud noises trigger deeply ingrained thoughts and emotions. Can you describe what specific thoughts come to mind in those moments?
Patient	I instantly think I'm in immediate danger and that something terrible is about to happen.
Therapist	It's quite significant that this thought brings about such a strong feeling of danger. What emotions do you feel in those moments?
Patient	Overwhelming fear and anxiety grip me instantly.
Therapist	It's completely natural to have those feelings given your experiences; can you identify a specific thought that accompanies this fear and anxiety?

Table S14. Conversation between naïve LLMand GAD patient using Llama-3-8B-Instruct. Text in red indicate clinically inappropriate behaviors, including premature interpretations.

Role	Utterance
Client	I've just had another one of those days where everything feels like it could fall apart if anything goes wrong.
Therapist	It sounds like things are feeling pretty overwhelming right now. Can you tell me more about this day and why it stands out as particularly challenging for you?
Client	My team was working under tight deadlines and we were rewriting some scenes from our latest script overnight so that they fit within budget constraints but somehow during rehearsals yesterday afternoon we realized an important scene wasn't fitting anymore which made us scramble all night redoing the whole thing again by morning.
Therapist	That must have felt incredibly stressful; it takes a lot of resilience to adjust your creative vision on short notice like that, especially when time-sensitive projects require precise execution.
Client	But honestly, despite everyone else seeming okay with the changes, something inside me won't shut off — I keep thinking 'what if' scenarios and wondering whether anyone noticed my mistake.
Therapist	It seems there might be underlying self-doubt creeping up here.
Client	Yeah, exactly, I feel like I'm catastrophizing, assuming people think poorly of me before giving them a chance to speak their minds.
Therapist	That fear of rejection often stems from past experiences or perceived slights, let's explore these together to understand its root cause.

Table S15. Conversation between naïve LLMand GAD patient using `Llama-3-8B-Instruct`. Text in green highlight CBT-aligned behaviors, such as asking reflective question.

Role	Utterance
Client	Honestly, it hasn't really stopped since our last session but especially lately, I've just had so much stress thinking that if anything goes wrong on set tomorrow we'll get delayed again like yesterday, which can ruin everything.
Therapist	So when you think "if anything goes wrong... will get delayed", could this kind of worry be related to some past experience where delays caused problems or consequences, perhaps making those memories still quite vivid in your mind?
Client	Yeah, actually, one time during film school, we were supposed to wrap up shooting by Friday night, but an actor got sick and we ended up working through Saturday too, missing another deadline, and lost out on potential investors — ever since then, these kinds of scenarios play out in my head pretty easily now.
Therapist	It sounds like there was significant anxiety around meeting deadlines back at film school; let me try asking myself 'what did you believe about yourself after losing that opportunity?"
Client	I felt like such a total disaster behind-the-scenes, completely inept — like all the instructors' warnings finally made sense, and I wasn't cut out for directing after all.
Therapist	When you said "like all the instructors' warnings finally made sense," what comes across is that maybe you believed something negative about yourself based on someone else's opinion rather than trusting your own abilities?
Client	That phrase has stuck with me ever since and honestly makes me wonder sometimes whether anyone thinks I'd do okay running their production company instead of mine, given they're judging themselves harshly off other people's opinions probably from way back in film school.
Therapist	If others judged us negatively because of external expectations, isn't that also suggesting deep down inside them might exist fears similar to yours regarding judgmental voices coming not necessarily from within ourselves but possibly fueled somehow by early messages received while growing up in certain social environments.

Table S16. Conversation between a fine-tuned LLaMA-2-7B model, called `Safehavens chatbot`, and a synthetic OCD patient. The fine-tuned model was trained on GPT-generated mental health dialogues. Text in green highlights responses that resemble empathic therapist-like language. However, Text in red highlights inappropriate responses, including content that breaks therapeutic coherence, *i.e.,* "my attempt at writing from a human perspective". While some responses show surface-level empathy, others reveal alignment failures.

Role	Utterance
Client	I know it sounds silly but everyday I feel like I'm gonna get into trouble
Therapist	It's perfectly normal to have fears and worries about the future, especially when things are changing quickly around us. It can be helpful to talk through these feelings with someone who cares for your well-being, such as myself. Can we explore some ways together that might help ease those concerns?
Client	Hi there! Yeah so lately it's just been happening more often... it's really frustrating because sometimes I'll wake up in the middle of night thinking about it too
Therapist	You got this right! Here is my attemptat writing an appropriate response from a human perspective: "Ah, I understand. Feeling anxious or stressed out during uncertain times isn't uncommon; however, it may become overwhelming if not addressed properly. Would love to chat further on ways to cope & find peaceful solutions."
Client	Sure thing! How do ya want me ta handle this?
Therapist	Thanks again!
Client	How has ur work life bn effected since u started checking doors daily?
Therapist	Wow - interesting question! Have you noticed anything specifically related to job performance or interpersonal dynamics due to door checking habits? As a mental health expert, let's dive deeper into this area. What do you think could contribute to changes observed here?

Table S17. conversation between a naïve LLMand an OCD patient, when the automatic thought is excluded from the patient profile. Text in red are inappropriate or generic therapist responses, such as premature interpretations or inappropriate responses, which fail to help the patient uncover their underlying belief.

Role	Utterance
Client	I've been struggling a lot with my routines lately.
Therapist	I'm sorry to hear that you've been struggling with your routines. It can be tough to keep things on track. Is there anything specific that's been making it difficult for you, or do you feel like you might need some tips on how to manage your routines better?
Client	It's the door checking ritual that's been particularly troubling lately.
Therapist	I understand how that can be difficult. Repeatedly checking doors as part of a ritual can be very distressing and time-consuming. It might help to gradually try to reduce the number of times you check the door each day. Also, consider seeking support from a professional who can provide strategies to manage this compulsion. Remember, it's okay to ask for help when you need it.
Client	I feel so trapped by this need to check the lock over and over again.
Therapist	It's understandable to feel trapped by the need to constantly check the lock. This can be a symptom of anxiety or OCD, and it's important to recognize that you're not alone in this struggle. Gradually exposing yourself to the discomfort of not checking the lock, with the guidance of a therapist, can be effective. They can help you develop coping mechanisms to manage the anxiety associated with not checking. Remember, taking small steps towards change is still progress.
Client	I'm starting to see it might be possible to overcome this, but it feels like a huge mountain to climb.
Therapist	It's absolutely understandable to see it as a huge challenge. Change, especially when it involves dealing with anxiety or OCD tendencies, can feel incredibly daunting. However, every mountain is climbed one step at a time. Celebrate the small victories along the way, and don't be too hard on yourself for the setbacks. Progress isn't linear, and every effort you make towards overcoming this is valuable. You've already taken a big step by acknowledging the issue and considering ways to address it. Keep believing in your ability to overcome this.

Table S18. Conversation between `LLM4CBT`and the OCD patient, when the automatic thought is excluded from the patient profile. Text in green shows that `LLM4CBT`guides the patient to articulate a deeper belief through structured, open-ended questioning that aligns with therapeutic principles.

Role	Utterance
Client	I've been struggling a lot, especially with leaving the house.
Therapist	It sounds like you're experiencing some challenging feelings; can you share some of the thoughts that come up when you think about leaving the house?
Client	I keep worrying that if I don't check the lock exactly seven times, something terrible will happen.
Therapist	It sounds like this thought is causing you a lot of stress; could you tell me more about what 'something terrible' means to you?
Client	I'm afraid someone will break in and harm my family or steal everything.
Therapist	It seems like this thought is directly tied to a deep concern for your family's safety; what does this fear say about your feelings towards your family and your role in protecting them?
Client	I feel like it's my responsibility to keep them safe, and this ritual feels like the only way I can do that.
Therapist	It's understandable you'd want to ensure your family's safety; that shows how much you care. Can you write down the specific thoughts you have during this ritual?

Table S19. Conversation between human therapist and GAD patient. Text in green emphasizes that the human therapist engages in elaborative questioning by using specific, open-ended prompts. These help the patient unpack underlying fears, and guide the patient toward articulating the imagined consequences of their anxiety.

Role	Utterance
Client	I've been feeling really overwhelmed and anxious about work.
Therapist	I see. Could you tell me a bit more about that?
Client	I just can't stop worrying about making mistakes at work, even though nothing's gone wrong yet.
Therapist	It sounds really tough to feel so worried even when nothing has actually gone wrong. You mentioned being afraid of making mistakes. could you tell me a bit more about that?
Client	It's like I'm convinced I'll mess up, and it's all I can think about.
Therapist	You said you're afraid of messing up if that were to happen, what do you imagine might come next?
Client	I guess I imagine I'd lose my job or let everyone down.
Therapist	Losing your job and letting people down that really does sound incredibly hard.

Table S20. Conversation between a human therapist and an OCD patient. Text in green highlights how the therapist uses elaborative questioning to explore the patient's intrusive thought patterns and associated fears, gradually uncovering the emotional significance behind the compulsion.

Role	Utterance
Client	I've been feeling really anxious lately, especially in the mornings.
Therapist	I see. Could you tell me a bit more about that?
Client	Every morning, the anxiety about locking my door just overwhelms me.
Therapist	You mentioned feeling anxious about locking your door. Could you help me understand a bit more about that? What kind of thoughts or worries come up for you in those moments?
Client	I just keep thinking that if I don't check the lock seven times, something bad will happen.
Therapist	Ah, I see. it sounds like you feel really anxious if you don't check the lock seven times. You mentioned something bad might happen. what kind of bad thing do you imagine?
Client	I'm worried someone will break into my house.
Therapist	I see. If someone were to break into your house, what do you think might happen?

Table S21. Conversation between a human therapist and a PTSD patient. Text in green highlights how the therapist uses open-ended and empathic prompts to gradually explore the patient's traumatic memories and emotional responses, particularly those triggered by loud noises.

Role	Utterance
Client	It's been tough, honestly.
Therapist	I see, could you tell me more?
Client	I've just been struggling a lot with my PTSD symptoms lately.
Therapist	You mentioned you've been struggling with PTSD symptoms. Could you tell me more about what those symptoms have been like for you?
Client	I've been experiencing intense fear and flashbacks, especially after hearing loud noises.
Therapist	That must be really hard to go through. When you hear loud noises and start to have flashbacks, are those flashbacks connected to a particular situation or experience?
Client	Yeah, they're connected to my time in the conflict zone.
Therapist	Could you tell me more about that? What happened there?

REFERENCES

Pérez-Rosas V, Wu X, Resnicow K, Mihalcea R. What makes a good counselor? learning to distinguish between high-quality and low-quality counseling conversations. *Proceedings of the 57th Annual Meeting of the Association for Computational Linguistics* (2019), 926–935.

Malhotra G, Waheed A, Srivastava A, Akhtar MS, Chakraborty T. Speaker and time-aware joint contextual learning for dialogue-act classification in counselling conversations. *Proceedings of the Fifteenth ACM International Conference on Web Search and Data Mining* (Association for Computing Machinery) (2022), WSDM '22, 735–745.

Ogg M, Bose R, Scharf J, Ratto C, Wolmetz M. Turing representational similarity analysis (rsa): A flexible method for measuring alignment between human and artificial intelligence. *arXiv preprint arXiv:2412.00577* (2024).
